# Catalytic mechanism of α-phosphate attack in dUTPase is revealed by X-ray crystallographic snapshots of distinct intermediates, ^31^P-NMR spectroscopy and reaction path modelling

**DOI:** 10.1093/nar/gkt756

**Published:** 2013-08-27

**Authors:** Orsolya Barabás, Veronika Németh, Andrea Bodor, András Perczel, Edina Rosta, Zoltán Kele, Imre Zagyva, Zoltán Szabadka, Vince I. Grolmusz, Matthias Wilmanns, Beáta G. Vértessy

**Affiliations:** ^1^Laboratory of Genome Metabolism, Institute of Enzymology, Research Center for Natural Sciences, Hungarian Academy of Sciences, Budapest H-1113, Hungary, ^2^Laboratory of Molecular Biology, NIDDK, NIH, Bethesda, MD 20892, USA, ^3^Structural and Computational Biology Unit, European Molecular Biology Laboratory, Heidelberg D-69117, Germany, ^4^Laboratory of Structural Chemistry and Biology, Institute of Chemistry, Eötvös Loránd University, Budapest H-1117, Hungary, ^5^Protein Modelling Group MTA-ELTE, Institute of Chemistry, Eötvös Loránd University, Budapest H-1117, Hungary, ^6^Department of Chemistry, King's College London, London, SE1 1UL, UK, ^7^Department of Medical Chemistry, University of Szeged, Hungary, ^8^Department of Computer Science, Eötvös Loránd University, Budapest, Hungary, ^9^European Molecular Biology Laboratory, Hamburg Outstation, Hamburg D-22603, Germany and ^10^Department of Applied Biotechnology and Food Sciences, Budapest University of Technology and Economics, Budapest, Hungary

## Abstract

Enzymatic synthesis and hydrolysis of nucleoside phosphate compounds play a key role in various biological pathways, like signal transduction, DNA synthesis and metabolism. Although these processes have been studied extensively, numerous key issues regarding the chemical pathway and atomic movements remain open for many enzymatic reactions. Here, using the Mason–Pfizer monkey retrovirus dUTPase, we study the dUTPase-catalyzed hydrolysis of dUTP, an incorrect DNA building block, to elaborate the mechanistic details at high resolution. Combining mass spectrometry analysis of the dUTPase-catalyzed reaction carried out in 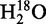 and quantum mechanics/molecular mechanics (QM/MM) simulation, we show that the nucleophilic attack occurs at the α-phosphate site. Phosphorus-31 NMR spectroscopy (^31^P-NMR) analysis confirms the site of attack and shows the capability of dUTPase to cleave the dUTP analogue α,β-imido-dUTP, containing the imido linkage usually regarded to be non-hydrolyzable. We present numerous X-ray crystal structures of distinct dUTPase and nucleoside phosphate complexes, which report on the progress of the chemical reaction along the reaction coordinate. The presently used combination of diverse structural methods reveals details of the nucleophilic attack and identifies a novel enzyme–product complex structure.

## INTRODUCTION

Nucleophilic substitution reactions on phosphoric acid derivatives (such as anhydrides or esters) are involved in metabolism, signal transduction and nucleic acid biosynthesis and processing. Whereas the γ-phosphate group of nucleoside triphosphates is quite reactive (e.g. ATP or GTP γ-phosphate), their α-phosphate position as well as the phosphodiester linkage in nucleic acid polymers are significantly more inert to prevent unwanted modifications (1–3). Reactions at these sites require powerful enzyme catalysts, e.g. nucleases, polymerases (4), Nudix hydrolases and dUTPases.

**Figure 1.**
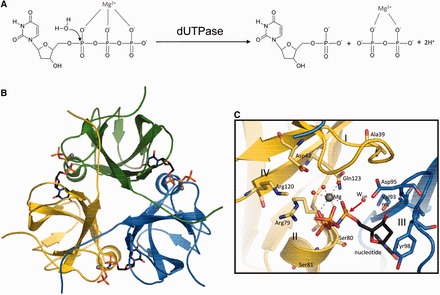
Nucleoside phosphate hydrolysis reaction catalyzed by dUTPase. (**A**) Reaction scheme of dUTP hydrolysis. (**B**) The overall architecture of the symmetric dUTPase homotrimer (M-PMV dUTPase complete E-S complex). Colour-coded subunits and α,β-imido-dUTP substrate in atomic colours (C: black, O: red, N: blue, P: orange, Mg: grey). The trimer has three active sites, depicted by the bound ligands, located on the bimolecular interfaces. (**C**) Close up of one dUTPase active site. Conserved catalytically important residues are shown with bonds. Protein carbons are in cyan or yellow reflecting the subunit colour; all other moieties are in atomic. Roman numbers I–IV: conserved dUTPase motifs: Motif I – residues 39–42, Motif II – residues 78–81, Motif III – residues 92–100, and Motif IV – residues 120–123. Residue numbering is for the dUTPase domain only (not including the N-terminal nucleocapsid domain unique to beta-retroviral dUTPases). Red arrow points out the direction of nucleophilic attack by the ordered water molecule (W_cat_).

The ubiquitous dUTPase enzyme catalyzes dUTP cleavage to dUMP and pyrophosphate (Figure 1A) (5). This reaction contributes to thymidylate biosynthesis by producing dUMP (6,7). Additionally, it strictly controls cellular dUTP/dTTP ratios and thus prevents uracil incorporation into DNA. Targeting enzymes of *de novo* thymidylate biosynthesis by fluorinated nucleotide derivates, methotrexate or other antifolates is a widespread approach in chemotherapy against cancer or diverse pathogenic microorganisms (8–11). These drugs perturb the cellular dUTP/dTTP pool and contribute to elevation of uracil levels in DNA. Uracil-substituted DNA transforms base-excision repair into a hyperactive cycle inducing DNA double-strand breaks and thymine-less cell death. In this respect, dUTPases, important for preserving genomic integrity (12), have been proposed as targets against cancer (13–18) and multiple infectious pathogens, as well (5,7,19–25). The potential use of dUTPase antagonism in inducing thymine-less cell death requires a detailed understanding of the enzymatic mechanism.

The prototypical β-retrovirus Mason–Pfizer monkey retrovirus (M-PMV) expresses its dUTPase as a bifunctional protein together with the retroviral nucleocapsid protein. The M-PMV dUTPase was shown to be fully functional, although somewhat less active (k_cat_ decrease by a factor of 10–20) than other dUTPases (26,27), most likely due to deletion of a short peptide segment in a non-conserved C-terminal region (26). In most dUTPases (including M-PMV), β-pleated subunits assemble into domain-swapped homotrimers (Figure 1) (cf (5,28) for reviews, also (29–33) for representative original reports). Three active sites are located in clefts between neighbouring subunits and recruit conserved residues from different subunits (Figure 1). Active site residues belong to one of the five conserved sequence motifs (Motif I–V, Figure 1C) (5,28). The conservation of the side chains in the active site in trimeric dUTPases is remarkably high, the amino acids accommodating the nucleotide ligand and the catalytic water are strictly conserved in all species, from viruses to man (5,6,28,34,35). Studies on human and bacterial dUTPases indicated that a water molecule, oriented by a conserved aspartate, initiates nucleophilic substitution at the α-phosphorus (αP) (Figure 1C) (31,36–38). These studies used a modified substrate, α,β-imido-dUTP (39), which has a significantly lower enzyme-catalyzed hydrolysis rate than the physiological substrate dUTP, although the binding geometries of the two substrates are identical (26,36). To our current knowledge, the major aspects of the catalytic mechanism are highly conserved among trimeric dUTPases [detailed mechanistic studies are available for dUTPase from *Escherichia coli* (36,40,41), equine infectious anemia retrovirus (32,42–44), *Bacillus subtilis* (33,38,45), human (30,37,46), *Drosophila melanogaster* (47,48) and *Mycobacterium tuberculosis* (25,31,49,50)]. This is especially true for the mechanism of nucleophilic attack, whereas differences are observed in the flexibility of the C-terminal arm [cf. (38)].

Current approaches to investigate enzyme mechanism at the atomic level [X-ray crystallography (4,51–53), ^31^P-NMR spectroscopy (54,55), *ab initio* QM/MM simulations (56–60), etc.] provide highly significant key observations, but with each having their inherent limitations requiring integration of multiple methods. In the present work, we have taken advantage of the low k_cat_ value of the M-PMV dUTPase (26) (Figure 1) and a suboptimal substrate, α,β-imido-dUTP (36) (cf. Supporting Data), to study the reaction mechanism in four dimensions. Here we combine enzyme kinetics, mass spectrometry, ^31^P-NMR spectroscopy, X-ray crystallography and theoretical calculations to analyse the molecular structures formed during the catalytic reaction of this essential enzyme. We use the M-PMV dUTPase for this study for two main reasons: (i) it could be crystallized fast and very reproducibly, to allow for either co-crystallization or soaking trials, and (ii) the catalytic rate constant of this enzyme is about 10-fold smaller than for other dUTPases, slowing down the reaction to facilitate observation of the different complexes. In the present study, we suggest a detailed route for the attacking nucleophile oxygen to approach the reaction centre. We also directly visualize a previously undescribed enzyme–dUMP complex where the product dUMP reflects a probably transient post-inversion conformer.

## MATERIALS AND METHODS

### Mass spectrometry

Hydrolysis of dUTP by dUTPase was followed in ^18^O-water (^18^O content > 92%). The reaction product dUMP was analysed by mass spectrometry. After isocratic HPLC (Applied Biosystems 140 C; methanol:water 10:90; flow rate: 250 µl/min; sample volume: 20 µl; column YMC J‘sphere H80 4 lm, 80 A, 50 × 2.1 mm and Phenomenex Luna 5 µm, 100A, 50 × 2.0 mm), samples were analysed on a Finnigan TSQ_7000 triple quadrupole mass spectrometer (Finnigan_MAT, San Jose, CA) equipped with a Finnigan Atmospheric Pressure Chemical Ionization source in positive ion mode using selective reaction monitoring. Standard dUMP (from Sigma) was measured as a control for product not containing ^18^O.

### ^31^P-NMR spectroscopy

^31^P-NMR spectra were acquired at 101.25 MHz on a Bruker Avance 250 spectrometer using a 5-mm inverse ^1^H/^13^C/^31^P/^19^F probehead. Chemical shifts were referenced to an external 85% H_3_PO_4_ standard (0.00 ppm), and all measurements were performed at 296 K. Reaction mixtures contained 0.2–0.8 mM α,β-imido-dUTP, 9 mM MgCl_2_, 50 mM Tris/HCl pH 8.0, 200 mM NH_4_Cl and 10% D_2_O. The enzyme was added subsequently, reaching a final ratio of α,β-imido-dUTP:dUTPase = 1:1. Quantitative spectra were obtained by applying inverse gated proton decoupling sequences. A 5-µs-long 30° pulse was used, total relaxation delay was 2.0 s and the number of transients varied between 4600 and 21 000, depending on sample concentration. Data were analysed with the software package XWINNMR. Integrated intensities obtained from quantitative data collection were fitted simultaneously for all six peaks assuming first-order kinetics with equation a = a0*exp(-k*t) for the increasing and b = b0*(1-exp(k*t)) for the decreasing points.

### Structure determination and refinement

Twelve crystal structures were solved (Table 1, Supplementary Table S1). One structure containing a mixture of complexes of dUTPase with substrate and a post-inversion product conformer (PDB ID 3TPY) was solved by experimental phasing using a platinum derivative (61). Two platinum sites were found, and phasing was carried out using Single Isomorphous Replacement with Anomalous Scattering in the SOLVE/RESOLVE package (62). The good-quality experimental phases (mean figure of merit: 0.405) (61) provided a readily interpretable electron density map allowing automated model building using ARP/wARP for residues 23–136. The map calculated from this initial model revealed strong density for the nucleotide ligand. The refined 3TPY structure was used in a previous study to solve an unliganded C-terminally truncated mutant M-PMV dUTPase structure [PDB ID: 2D4L (26)] by direct rigid body refinement. All the subsequent structures of the current study were solved by rigid body refinement using the latter unliganded dUTPase structure (2D4L) as a starting model. By using an unliganded enzyme structure that was refined thoroughly against the data collected on the apo-enzyme crystals, we sought to minimize model bias with respect to ligand position. In all cases, the resulting initial maps were of high quality, allowing residues 1–136 and entire ligands to be built with ease. The asymmetric unit contained one subunit.

**Table 1.** List of structural snapshots

Group	Structure identifier (PDB ID)	Mode of preparation	Resolution Å	Information content
E	2D4M (36)	Crystallization of apo enzyme in the absence of nucleotide ligands.	1.83	W_cat_ bound in the apo enzyme.
E-S	3TPN	Soaking the slow substrate α,β-imido-dUTP into apo crystals.	1.65	Substrate diffused into the active site. W_cat_ is in proximity to the nucleotide substrate. Figures 1C and 3B and 4B
	3TPS		1.85	
	3TP1	Relatively long incubation of complex crystals in excess of the slow substrate α,β-imido-dUTP.	1.6	
E-S/E-piP mix	3TPY	Co-crystallization of enzyme–substrate complex (dUTPase and the slow substrate α,β-imido-dUTP was mixed in solution before crystallization).	1.75	Chemistry completed in part of the active sites. Figures 3C and 4A, and B
	3TQ3		1.85	
	3TQ4		1.6	
E-piP	3TQ5	Incubation of complex co-crystals (grown from a mixture of dUTPase and the slow substrate α,β-imido-dUTP) for over 4 days.	1.4	Chemistry completed throughout the crystal. Inversion directly visualized. Figures 3D and 4C and D
	3TRL		1.8	
	3TRN		1.83	
E-P	3TS6	dUMP soaking into apo crystals.	1.84	Stable conformation of the product dUMP. Figures 3E and 4D
	3TTA		2.0	
	3TSL	Crystallization of enzyme dUTP complex (dUTPase and dUTP was mixed in solution before crystallization).	2.2	Conformation of the end-product of the physiological reaction.

Generation of subunit libraries for the ligands (α,β-imido-dUTP and dUMP) and refinement were carried out using CCP4i and Refmac5 (63,64). Positional and B-factor refinement rounds were alternated with manual rebuilding steps using the graphics programs Coot (65) and O (66). ARP/wARP (67) was used for water building. Refinements of the enzyme–nucleotide structures containing the substrate, several intermediates or the product were pursued with restraining substrate-like nucleotide geometry. At late stages of the refinement, as the anomaly of the ligand structure became clear, the library files were manually edited to permit larger deviation for the affected bond distances and angles. This allowed the ligand to be properly accommodated in the observed electron density. Residues belonging to the C-terminal Motif V (residues 137–152) are hardly visible even in the final maps and are therefore mostly omitted from the model. A summary of the crystallographic data collection and refinement statistics is given in Supplementary Table S1.

Figures were produced using Pymol (68). Simulated annealing composite omit electron density maps (sigmaa-weighted 2Fo-Fc maps) were calculated using CNS v1.1 (69). Coordinates and structure factor data have been deposited in the Protein Data Bank (Table 1, Supplementary Table S1).

### Reaction path modelling

The initial structure was created based on the complete enzyme–substrate (E-S) structure as follows: The coordinates of the C-terminal arm (not visible in the crystal structure) were modelled based on the structure of the human dUTPase [PDB ID: 2HQU (37)], as described previously (26). The N atom in the α,β-imido-dUTP ligand was replaced by oxygen to reproduce the wild-type substrate. Crystallographic water molecules in the vicinity of the protein or the substrate were preserved. This initial structure was then customized for QM/MM simulations using Molecular Dynamics following the protocol described in (70,71).We performed minimizations both in the forward and backward directions of the chemical reaction to obtain hysteresis-free results (72). In the QM/MM simulations, the equilibrated classical system was trimmed to a sphere of 31 Å radius centred on the Mg^2+^ ion of chain B. Residues farther than 20 Å from the Mg^2+^ ion were kept fixed. We used the Q-Chem software package to perform the *ab initio* density functional theory calculations, with the B3LYP (73) 6–31 + G(d) level of theory. The QM system was subsequently coupled with the CHARMM program using full electrostatic embedding (74). The QM system consisted of 94 atoms in total, including the hydrogen atoms. The quantum region contained the Val93 and Ile94 backbones, the full Asp95 residue and the side chain of Arg141; the Ser80 side chain, and the amide group of Gln123 from the respective subunits; the Mg^2+^ ion with its full coordination shell; nearby crystallographic water molecules and the triphosphate and deoxyribose groups of dUTP.

## RESULTS AND DISCUSSION

### dUTPase-catalyzed cleavage of both dUTP and α, β-imido-dUTP involves nucleophilic substitution on the αP

Previously determined crystal structures of human and bacterial dUTPase–α,β-imido-dUTP complexes suggested nucleophilic attack of a catalytic water molecule (W_cat_) at the αP of the substrate (31,36,37). To directly investigate this mechanism of attack in the solution phase on M-PMV dUTPase, we followed the enzyme-catalyzed dUTP hydrolysis in ^18^O water and analysed the dUMP product by mass spectrometry. The molecular mass of control dUMP (not containing any heavy isotope) was measured to be 317 Da, whereas dUMP formed in the M-PMV dUTPase-catalyzed reaction in ^18^O water was measured to be 319 Da. This means that the heavy isotope water oxygen became bonded to the αP, hence the nucleophilic attack must have occurred at this site. The same experiment was also performed with the slow substrate α,β-imido-dUTP. This analogue, although imido-linkages are usually considered to be enzymatically non-hydrolyzable, was previously shown to be cleaved by dUTPase producing dUMP (36). The mass spectrometric experiment using α,β-imido-dUTP revealed a dUMP mass of 319 Da, confirming that αP attack also holds for α,β-imido-dUTP. These results indicate that the retroviral enzyme shares chemistry with other dUTPases: it initiates dUTP hydrolysis via nucleophilic attack of a water molecule on the αP.

### dUTPase-catalyzed α,β-imido-dUTP hydrolysis can be followed in solution by ^31^P-NMR

^31^P-NMR spectroscopy is a powerful technique to follow reactions at a phosphorus centre (55). The phosphorus atom features high gyromagnetic ratio, complete natural abundance, chemical shift values sensitive to coordination and scalar coupling values directly correlated with bond order. Hence, it is a sensitive probe to detect changes in the bonding environment of phosphate groups. The technique is also fast and non-invasive, facilitating kinetic studies *in situ*. In addition, the dUTPase–α,β-imido-dUTP system is especially well suited for time-resolved analysis by ^31^P-NMR because: (i) the phosphates in α,β-imido-dUTP are expected to show distinct chemical shifts; (ii) relative to these, the reaction products feature altered chemical shifts that also depend on the site of the nucleophilic attack and (iii) the reaction is slow (36) and can therefore be followed on the NMR time scale. Hence, we decided to analyse the progress of this reaction by ^31^P-NMR spectroscopy.

**Figure 2.**
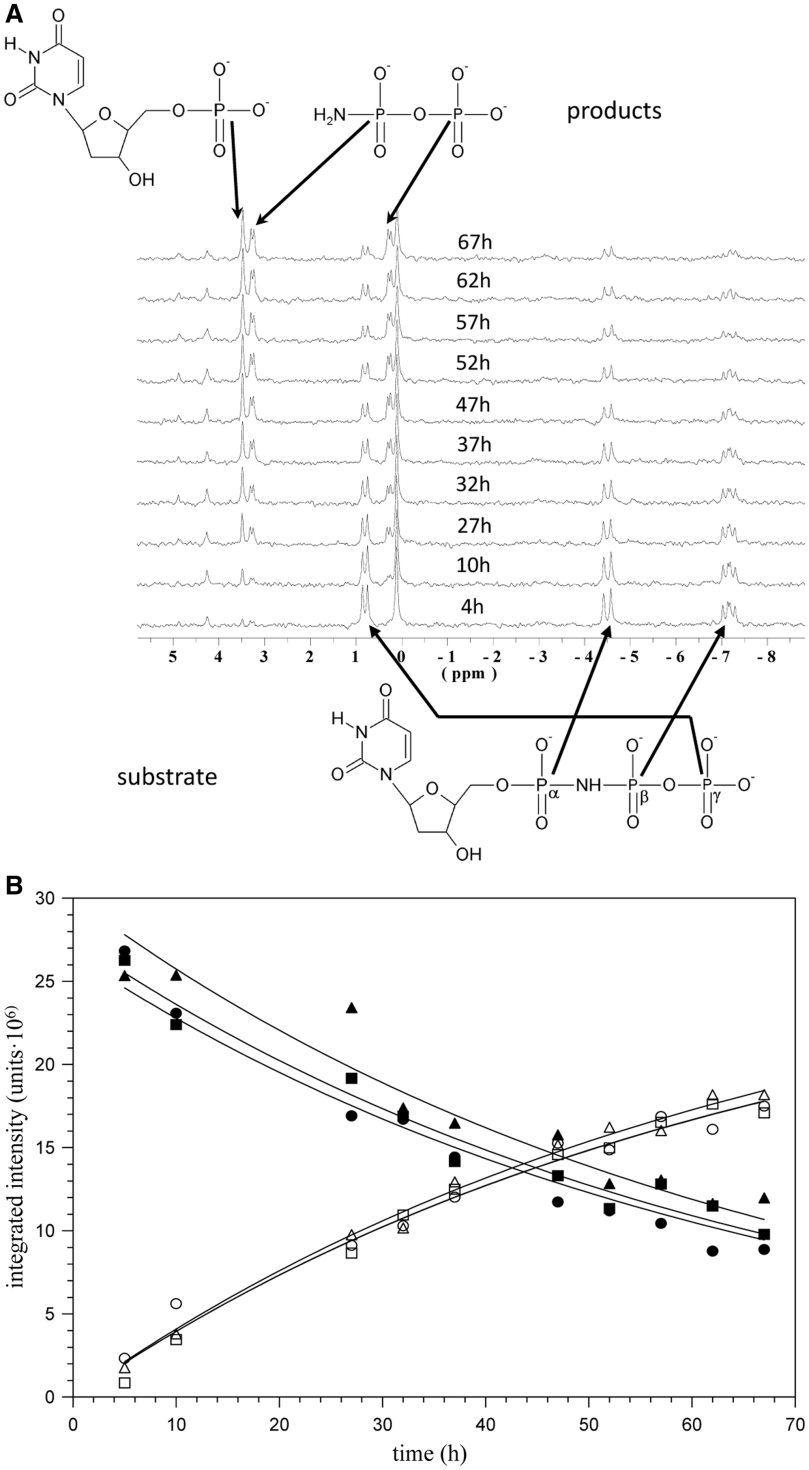
^31^P{^1^H} NMR spectra following progress of α,β-imido-dUTP hydrolysis catalyzed by dUTPase. (**A**) Spectra were recorded at different time intervals, phosphorus resonances of both the nucleotide substrate and products are highlighted. (**B**) Kinetic analysis of α,β-imido-dUTP hydrolysis as followed by ^31^P-NMR spectroscopy. Intensity variation of the resonances as a function of reaction time. Decreasing data sets of closed squares, triangles and circles correspond to the signals of α-, β- and γ-phosphorus atoms of α,β-imido-dUTP, respectively; increasing data sets of squares, triangles and circles correspond to the signals of phosphorus atoms of dUMP and inorganic pyrophosphate, respectively. Solid lines indicate results of simultaneous fitting of all data sets.

First, the ^31^P{^1^H} NMR spectrum of α,β-imido-dUTP was recorded (Figure 2A, bottom spectrum) and showed three peaks belonging to the three phosphorus atoms with characteristic couplings from the neighbouring phosphorus: α-P (−4.5 ppm, doublet, ^2^J_PP_ = 16.0 Hz), β-P (−7.16 ppm, doublet of doublet, ^2^J_PP_ = 16.0 and 10.8 Hz) and γ-P (0.8 ppm, doublet, ^2^J_PP_ = 10.8 Hz). An additional peak was found in the spectrum at 0.10 ppm (also present in the α,β-imido-dUTP stock solution) and was identified as phosphate impurity. On addition of the enzyme to α,β-imido-dUTP, no observable changes occurred in the first few hours, indicating that coordination of the phosphate oxygens by the enzyme does not perturb chemical shifts of the phosphorus atoms. However, when monitoring the mixture over longer time, new species appeared after 7–10 h: two doublets at 3.27 ppm and 0.27 ppm, both showing a 6.5-Hz coupling constant, and one singlet at 3.48 ppm (Figure 2A, upper spectra). The intensity ratio of these peaks was 1:1:1. Over time, the intensities of the new peaks increased, and, in parallel, the intensities of the original peaks of α,β-imido-dUTP decreased. As the most plausible and straightforward explanation for these observations, we suggest that the new peaks resulted from the reaction products: two doublets from amino-pyrophosphate and one singlet from dUMP. Accordingly, when extra dUMP was added to the sample at the end of the reaction, the 3.48-ppm singlet peak increased. In agreement with our mass spectrometry results, these ^31^P-NMR experiments show that the products of the M-PMV dUTPase-catalyzed α,β-imido-dUTP hydrolysis are dUMP and amino-pyrophosphate, so the nucleophilic attack must have taken place on the αP atom.

During the course of the reaction, NMR spectra were collected in quantitative mode, allowing quantitative kinetic analysis. For this, the integrated intensities of all six peaks were plotted against time and fitted simultaneously (Figure 2B), assuming first-order kinetics. The resulting pseudo first-order rate constant of k = 0.015 ± 0.0005 s^−^^1^ (equivalent to k_cat_ of 1.9 × 10^−^^5 ^s^−^^1^) is in good agreement with the k_cat_ value measured for the *E. coli* dUTPase for α,β-imido-dUTP hydrolysis (36). Hydrolysis of 1 mM α,β-imido-dUTP in the presence of 0.8 mM M-PMV dUTPase was completed in approximately 4 days.

### Three-dimensional structures of dUTPase and its complexes with nucleotides visualized by X-ray crystallographic snapshots

**Figure 3.**
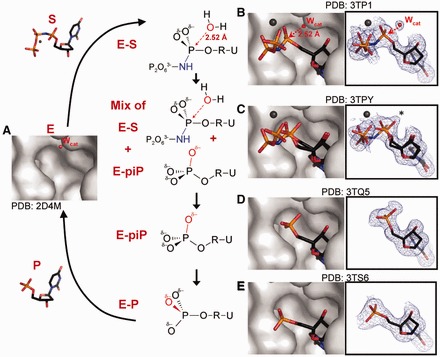
Structural snapshots. Schematics of distinct reaction intermediates are aligned with structural images captured in dUTPase-α,β-imido-dUTP complex crystals by X-ray diffraction. One representative structure is presented for each intermediary state. The apo-enzyme structure (**A**) reveals the binding site of the catalytic water molecule (W_cat_, red sphere). After binding of the substrate (**B**), we visualize a density map best described by a mixture of substrate and a novel product conformer [(E-S/E-piP mix structure (**C**)]. The temporary post-inversion conformation is also seen in the E-piP complex (**D**) that later relaxes to the end-product conformation [E-P complex (**E**)] by rotation of the α-phosphate group. On the schematics, U = uracil, R = 2′-deoxyribose, the nucleophilic attacker is shown as a red oxygen atom and the leaving group contains the blue NH moiety. The structural figures apply atomic colouring as in Figure 1C. Nucleotide ligands are shown as sticks, whereas for the protein only space filling surface is presented for clarity. In addition, simulated annealing 2Fo-Fc omit electron density maps are shown for reactants and products in representative structures at various sigma levels: E-S - 1.3σ, E-S/E-piP mix - 1.0σ, E-piP - 1.5σ and E-P - 1.7σ. Stereo images of the density maps are provided in the Supplementary (Supplementary Figure S2).

**Figure 4.**
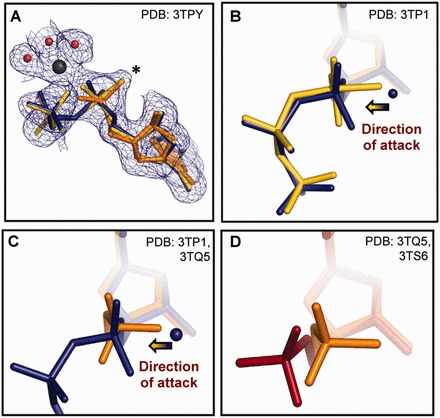
Close up of different ligand structures along the reaction pathway (**A**) Simulated annealed 2Fo-Fc omit electron density map calculated for the E-S/E-piP mix structure and shown at 1.0σ. The transition state mimicking intermediate model is shown with yellow sticks, and the mixture model is presented in blue and orange for the substrate (α,β-imido-dUTP) and post-inversion product (dUMP), respectively. Note that both models fit the density peak indicated with star similarly well, whereas the mixture model better explains the partial loss of density at the β- and γ-phosphates. (**B**) Superposition of the E-S (blue) and the transition-state (TS) mimic model (yellow) structures visualizes the linear approach of the nucleophile towards the α-phosphorus. Arrow indicates the direction of the nucleophilic attack. (**C**) Superposition of substrate (E-S, blue) and post-inversion product (E-piP, orange) complex structures highlights inversion on the α-phosphorus atom. Arrow points out the direction of the nucleophilic attack. D, Superposition of the two observed product conformations (piP: orange, P: red) reveals the rotation of the α-phosphate group that leads to relaxed end-product conformation.

**Figure 5.**
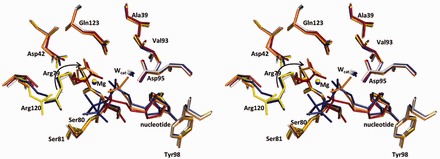
Protein side chains’ movements assist catalysis. Stereo view of the superposition of dUTPase active sites in all snapshots. Protein residues and the nucleotide ligand are shown with sticks representation, the Mg^2+^ ions with balls and the catalytic water molecules as stars. Colours follow from blue to red as the reaction progresses: E, white; E-S, blue; E-S/E-piP mix, side chains of the structure are in yellow, whereas S and piP are shown with blue and orange carbons, respectively; E-piP, orange; E-P, red.

In the present study, we have determined 12 crystallographic snapshot structures for dUTPase and its different complexes with nucleotides (Table 1, Supplementary Table S1, Figure 3). Optimization of crystallization for rapid growth resulted in well-diffracting crystals that grew in 3 days in the presence of α,β-imido-dUTP, and were analysed in the X-ray beam at various time intervals. To obtain structures at early stages of the reaction, we introduced apo-enzyme crystals into solutions containing α,β-imido-dUTP, and collected data sets after short incubation times. In parallel, we followed substrate conversion by analysing the drop composition with a discontinuous enzyme activity assay (27). In these, the overall protein fold and active site assembly are identical with previously reported structures (26,35,75) and also with each other. In the active sites, we observed four different nucleoside phosphate structures, which represent distinct reaction intermediates, as discussed later in the text (Figures 3–5).

### Complete E-S complex

By X-ray diffraction analysis of M-PMV dUTPase α,β-imido-dUTP complex crystals, we first captured the complete precatalytic complex containing enzyme and both reactants: the nucleotide and the catalytic water molecule (W_cat_) (Figures 3 and 4, Supplementary Figures S1 and S2). In this dUTPase–α,β-imido-dUTP–W_cat_ ‘ternary’ complex structure, the uracil and 2′-deoxyribose rings are accommodated by Motif III of subunit A. They sit in a β-hairpin held by H-bonding interactions with main chain atoms. The 2′-deoxyribose ring stacks against a conserved tyrosine (Tyr98 in M-PMV dUTPase), and its OH-group is H-bonded by the side chain of Asp95 (residue numbering is for the dUTPase domain only, not including the N-terminal nucleocapsid domain unique to beta-retroviral dUTPases). The correct localization of the uracil ring is supported by hydrophobic interaction with Ile94. Phosphate chain recognition is provided by conserved Motifs I, II and IV of subunit B. Motif V (residues 141–150) is not localized in the M-PMV dUTPase structures. However, independent molecular modelling and site-directed mutagenesis data indicate that it superposes onto the binding pocket and contacts the phosphate chain (26). Furthermore, the Mg^2+^ cofactor also coordinates to the three phosphates, and its coordination sphere is completed by water molecules that contact the side chain of Asp42 in Motif I.

The catalytic water molecule is precisely in the same coordination environment that was found in the *E. coli* and human enzymes (36,37), where the P-O distance varies from 3.6 to 2.8 Å. It is located near the αP opposite from the amino-pyrophosphate moiety in the extension of the scissile αP-N bond, and it is coordinated by the Val93 main chain carbonyl and the side chain of the strictly conserved Asp95. Interestingly, the distance between the αP and the W_cat_ oxygen (O_w_) (2.52 Å) is significantly shorter than the sum of the van der Waals radii of the two atoms [r_vdW_(P) + r_vdW_(O) = 3.32Å] [Figure 3B, cf. Ref. (37), see also Supplementary Figure S1]. We propose that this structure reflects the precatalytic stage of the reaction, where one water oxygen (O_w_) is already engaged in nucleophilic attack on the αP with in-line geometry. In the structure presented in Figure 3, the oxygen atom of this water molecule has a well-defined electron density that is clearly separated from that of the ligand or any other density in the structure, arguing that it originates from a separate entity, most likely water.

Although the precatalytic complex is a transient species, it can often be trapped by introducing modifications on either the substrate or the enzyme. Consequently, its structure has been determined for many enzymes (76,77), and has proven to be invaluable for understanding substrate binding and active site architecture and offers implications regarding the mechanistic pathway as well. For example, the precatalytic complex structure of DNA polymerase β (76) (Supplementary Figure S3) has resolved a long confusion about proper active site geometry and explained fidelity strategies applied by the enzyme. In addition, based on the distance between the nucleotide αP and the -OH nucleophile observed in the structure, an associative-like reaction mechanism could be proposed. We now capture the complete precatalytic complex of dUTPase due to an oxygen to nitrogen replacement in the substrate’s scissile bond. Interestingly, this modification does not abolish nucleotide hydrolysis (as shown by mass spectrometry and ^31^P NMR earlier). Therefore, we propose that this E-S complex structure represents a precatalytic complex structure that is not dead-end, and closely reflects the precatalytic state of the physiological reaction*.*

Further examples for short non-bonded P-O pairs are also available. For instance, in a ligand-bound structure of the acetylglutamate kinase (78) (Supplementary Figure S3), the authors have visualized the reacting phosphorus and oxygen atoms at a short distance with connecting electron densities. We have analysed enzyme structures in the Protein Data Bank and measured distances between non-bonded phosphorus and oxygen atoms (cf Supplementary Data). We plotted all occurrences of each P-O distance (Supplementary Figure S4) and show that roughly 90% of non-bonded P-O pairs are 3.3–4.5 Å apart, whereas the remaining pairs of P-O atoms are considerably closer to each other than the sum of van der Waals radii of the two atoms.

### Mixed populations of E-S and a suggested novel enzyme–product complex (mix of E-S and E-post-inversion-P mix)

Three crystallographic snapshots (see also Supporting Data) feature unexpected electron density within covalent bonding distance from the αP, close to the site of the catalytic water molecule in the E-S structures [star (*) position in Figures 3C and 4A]. In the crystallographic refinement, first we modelled substrate in the active site and restrained its geometry, but the α-PO_3_ moiety turned flat against the restraints, and the extra density became more and more prevalent. This called for a better interpretation of the electron density maps. Interestingly, there was no additional density near the α-phosphate that could be attributed to W_cat_, hence the appearance of this density in this structure was concomitant with the disappearance of the density associated with W_cat_ in the complete E-S structures.

Moreover, analysis of the refined electron density maps and phosphorus anomalous differences revealed that β- and γ-phosphates were less abundant in the structure than the α-phosphate. Consistently, refinement with full β- and γ-phosphate occupancies yielded high B-factors. Therefore, to improve the interpretation of the experimental data, we chose full occupancy for the uracil, deoxyribose and α-phosphate moieties and partial occupancy for the amino-pyrophosphate moiety (70–60% refined). As crystallography provides an averaged image over many copies of a molecule, partial occupancy suggests that the amino-pyrophosphate moiety is present in some but not all active sites in the crystal. This is possible if in some active sites, the catalytic reaction has already gone to completion and left dUMP behind. Such a mixture model (blue substrate and orange product in Figure 4) can well explain the development of the extra density on the αP (star position Figures 3C and 4A), assuming that this new position is occupied by one of the dUMP phosphate oxygens (orange in Figure 4A). Therefore, we next superposed published dUTPase–dUMP complex structures (31,36,79) on the observed electron density and found that the product dUMP has always been observed in a configuration where the star position is unoccupied. Despite the ambiguity regarding dUMP configuration, the mixture model provides a feasible and satisfying description of the density maps (perhaps preferable over the previously proposed intermediate compound (51), Figure 4A). Therefore, we wondered if the dUMP may assume an alternate configuration in the course of the M-PMV dUTPase catalyzed reaction, one that could account for densities left unexplained by previous structures. In the following text, we present this transient dUMP conformer.

### Transient post-inversion product conformer (E-piP): confirmation for the novel piP structure

In the next representative structure, we do not observe density for either the Mg^2+^ ion or the β- and γ-phosphates (Figures 3D and 4). The active site contains dUMP, suggesting that the reaction has been completed. Interestingly, the α-phosphate is observed in a novel configuration compared with previously published dUTPase-dUMP complex structures. One α-phosphate oxygen (O_w_) is located on the original line of nucleophilic attack, as if it was just introduced onto the α-phosphorus in the reaction from the attacking water molecule (Figure 4C). This structure agrees well with the notion of in-line attack and directly visualizes inversion on the αP. Superposition of this structure onto the E-S/E-piP mix structures and density maps shows the O_w_ atom overlapping with the ‘star position’ (Figure 4A) and resolves the aforementioned ambiguity regarding dUMP configuration.

### Relaxed end-product conformer (E-P)

Soaking apo M-PMV dUTPase crystals with solutions containing excess dUMP resulted in a structure that shows clear density for dUMP (Figure 3E). The conformation closely resembles the one seen in the *E. coli* dUTPase–dUMP complex structure (36). Compared with our E-piP structures, the phosphate configuration observed here is significantly different: it is shifted by 1.57 Å and rotated ∼40° around the O_5’_-αP bond (Figure 4D). Comparison of the interactions of the dUMP phosphate group with the enzyme environment in the E-piP and E-P structures shows that in the E-P structure, the phosphate group forms one additional H-bond and more favourable interactions with two other protein residues when compared with the E-piP structure. This suggests that the phosphate configuration observed in the E-P structures is more stable (see also Supporting Data) and hence is likely favourable over the piP conformation. We propose that the E-piP structure occurs in the reaction at an earlier stage than E-P. E-piP forms immediately after the scissile bond is broken and shows a nascent αP configuration; the α-phosphate is then rotated into the more stable end-product configuration (see also Supporting Data). Based on the series of structures discussed earlier and the apo-enzyme structure (PDB ID 2D4N), we simulated the reaction in an animation (Supplementary Movie S1) via morphing to illustrate the process of dUTPase-catalyzed nucleotide hydrolysis.

### Transition-state model also provides a good fit into the electron density of the structure representing the mixture of E-S and E-piP complexes

In some cases, direct experimental observations of transition-state (TS) structures have been claimed in the literature (51,80), but these immediately met intense debate (81). The authors of one report (51) suggested crystallographic trapping of the TS in the phosphoglucomutase reaction (Supplementary Figure S3) due to increased TS half-life in the enzyme (82) crystals. Electron density maps and other supporting data appeared convincing, although the possibility of TS mimicry by MgF3- ions (present in the crystallization solution) (81) has not been fully excluded. In our present E-S/E-piP mix structure, we captured a unique image (Figure 4A) that is in many ways reminiscent of the predicted TS for dUTP hydrolysis on the α-phosphate. Based on accompanying structures and theoretical calculations in this study, we conclude that the E-S/E-piP mix electron density is a result of density averaging over the crystal volume. Importantly, however, QM/MM simulations show (see later in the text) that the obtained image also closely mimics the TS structure of this hydrolysis reaction (cf yellow TS model in Figure 4A and B).

### Side chain conformational changes facilitate dUTPase catalysis

The specific role of protein dynamics in enzyme catalysis is currently under intense debate (72,83,84). Conformational motion is clearly essential for biological function, including enzyme catalysis (85). Numerous examples demonstrated biologically active and inactive conformations that are adopted by most biomolecular complexes, for example, to allow substrate binding or product release. However, a major question concerns whether specific protein motions are essential for the catalytic reaction. It is important to understand whether these conformational changes take place simultaneously with the catalytic reaction, or whether we can think about a catalytic complex as a stochastic, but relatively rigid, environment with preoriented protein groups that help activate the chemical step. We are now able to study a catalytic reaction with a modified substrate that allows us to sufficiently slow down only the chemical step, and therefore to observe experimental structural snapshots of the reaction underway. From these data we are able to pinpoint structural changes that occur within the time immediately before and right after the chemical step in the dUTPase enzyme.

To analyse how specific dUTPase residues contribute to nucleoside phosphate hydrolysis and explain the catalytic power of the enzyme, we performed multiple alignments of our structural snapshots (Figure 5). We found that the uracil and 2′-deoxyribose coordinating residues (β-hairpin, Tyr98, Ile94, Asp95—numbering for dUTPase domain only) remain mainly fixed throughout (Figure 5 and Supplementary Table S2). These residues occupy the exact same locations even in the apo-enzyme structure, suggesting that the bottom of the substrate binding pocket is rather rigid and is predesigned for stable binding of 2′-deoxiuridine. By fixing the conformation of the nucleoside moiety in this rigid pocket, dUTPase can position the α-phosphate precisely for nucleophilic attack. In our snapshot series, all molecular motions concern the phosphate chain and the residues involved in its coordination. Details of these movements and their possible roles in catalysis are discussed in the following text.

#### Asp95

The catalytic water molecule (Wcat), which initiates the reaction by nucleophilic attack, has been identified in the *E. coli* dUTPase structure and was later located in a number of structures from various sources (15,25,26). In the M-PMV dUTPase—similarly to other dUTPases—Wcat is coordinated by two protein residues: the carboxyl side chain of the conserved Asp95, and the main chain oxygen of Val93 (Figure 1C). The Asp95 side chain is held in place by its interactions with the 3′OH of the ribose ring and main chain NH of Ala39. This interaction network creates a well-defined binding site for Wcat.

**Figure 6.**
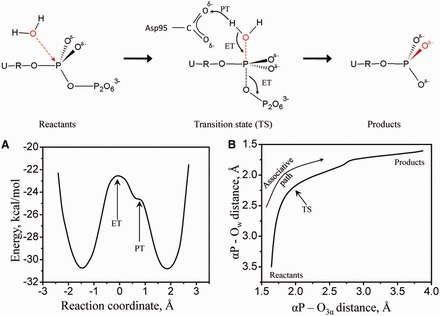
QM/MM simulations. Schematics of the simulated reaction with the chemical step (ET) and proton transfer (PT) events indicated. (**A**) Potential energy of the hydrolysis reaction by M-PMV dUTPase obtained from QM/MM minimizations along reaction coordinate Q2. The results indicate a synchronous transition state (bond breaking and forming at the phosphorus centre, ET) and a proton transfer step (PT) from W_cat_ to Asp95 immediately following the chemical step. (**B**) More O’Ferrall–Jencks plot showing the reaction path in the plane of the bond breaking and bond forming distances obtained by QM/MM minimizations along Q2. The QM region is also shown with the transition state structure.

On nucleotide binding, the Asp95 side chain is first rotated by 15.8° to reach for the 2′-deoxyribose 3′OH (Figure 5). This twist results in 0.61 Å shift in the position of the Asp95 Oδ2. In the nucleotide-bound conformation of Asp95, both of its carboxylate oxygen atoms are in H-bonding proximity of Wcat. This is in contrast to the apo-enzyme structure, where only Oδ1 contacts the Wcat. During the catalytic process, the Asp95 side chain shifts slightly to follow the Wcat towards the αP during the nucleophilic attack (Figure 5). This slight movement ensures that coordination of the attacking oxygen by Asp95 is preserved during the attack, maintaining activation of the nucleophile all the way through. The escorting behaviour of Asp95 is also in agreement with the results of our QM/MM simulations, suggesting that proton transfer from Wcat to Asp95 follows the chemical step (Figure 6). For this proton transfer, a direct contact between the Wcat and Asp95 must be maintained until after the chemical step.

#### Asp42

It is the only protein residue involved in coordination of the Mg^2+^ ion, which is primarily bound by the three phosphate groups of the substrate. During the reaction, Asp42 shifts within a 1.2 Å range (measured on Cγ, Figure 5). In the apo-enzyme structure, Asp42 turns away from the ligand binding pocket. On substrate binding, Asp42 turns to coordinate the Mg^2+^ by providing interactions for two of the water molecules in the coordination sphere of the Mg^2+^ ion. This later conformation is preserved in all nucleotide triphosphate–Mg^2^^+^-containing structures (E-S and E-S/E-piP mix). Finally, in both types of product complexes (E-P, E-piP), Asp42 obtains a similar conformation like in the apo enzyme. This is consistent with the lack of Mg^2+^ in all these structures, as this conformation would only allow coordination of one of the water molecules within the Mg^2+^ coordination sphere.

#### Phosphate coordinating residues

The main chain NH of Ser80 and the side chains of Gln123 and Ser80 form hydrogen bonds with the α-phosphate and play a key role in activation of the αP for accepting nucleophilic attack. They probably also stabilize the developing charge in the transition state. These residues encounter slight shifts (Figure 5) during the reaction, following movements of the α-phosphate oxygens. As the location of the α-phosphate is well preserved throughout the reaction, it is not surprising that we observe only small-scale protein rearrangements in its coordination sphere as well.

Ser 81, Arg79 and Arg120 reach towards the β- and γ-phosphates and contribute to binding and charge distribution of the phosphate chain. The main chain NH and side chain Oγ of Ser81 contact one of the β-phosphate oxygens. These interactions are not affected by the progress of the reaction (Figure 5). Arg79 and Arg120 side chains are mainly disordered in the apo-enzyme structure and become (partly) visible on substrate binding. Arg120 forms only water-mediated contacts with the γ-phosphate, and is, at least partially, disordered in 10 of the 12 structures (except one E-S and two E-S/E-piP mix structures). This suggests that it might not be essential for the whole process. Tackling the exact role of Arg120 in catalysis will require site-directed mutagenesis experiments.

We found that in the vicinity of the nucleotide ligand, Arg79 is the most flexible side chain (Figure 5). Arg79 is mainly disordered in the apo-enzyme structure and becomes (partly) visible on substrate binding, where it contacts both the β- and γ-phosphates with its Nε and NH2 atoms, respectively. In the product complex structures, it obtains an altered conformation and finds a location that was previously occupied by the Mg^2+^ in the E-S complex structures (Figure 5). The ability of Arg79 to occupy the position of the metal ion suggests that this residue might act as a metal-ion supplement for catalysis in metal-free conditions. This may explain the unique ability of dUTPases to hydrolyze the scissile bond with no strict requirement for divalent metal ions (1,4,27).

The last step of dUTP hydrolysis is the rotation of the dUMP α-phosphate from the post-inversion conformation (piP) to the end-product conformation (P). This movement is facilitated by interactions with the Arg79 side chain and the Ser80 main chain NH, which become stronger as a result of the E-piP to E-P transition. In addition, a dominant interaction with Ser80 Oγ is gained, whereas only weak contacts with Asp95 and Val93 are lost (Figure 5). Overall, these interactions make the α-phosphate orientation observed in the E-P structure preferable above the post-inversion configuration and drive the E-piP to E-P transition.

The E-piP complexes were obtained by incubation of complex co-crystals (grown from a mixture of dUTPase and the slow substrate α,β-imido-dUTP) for over 4 days. In the course of the in-line nucleophilic substitution mechanism, supported by our present study and also from other observations of the catalytic water (31,36,37), the incoming oxygen will form a covalent linkage with the alpha-phosphorus. After cleavage of the scissile bond, the transient post-inversion product is formed, which is then rotated into the relaxed product conformer. Although our present data do not provide direct evidence for this rotation, its occurrence can be presumed based on the identified reaction path and the observation of product conformers enzyme–product complex structures.

### Modelling the energetic pathway of dUTP hydrolysis

To model dUTP cleavage by dUTPase at the atomic level we performed QM/MM minimizations. For this, we first chose the reaction coordinate as the difference between the forming and breaking bond distances: Q1 = d(αP − O_3α_) − d(αP − O_w_). Using Q1, we could not observe a low-energy product state, and the hydrogen atoms of the attacking water molecule remained bonded at the end of the reaction. To resolve these issues and drive O_w_ deprotonation, we sought to incorporate proton transfer in the reaction coordinate. Investigations of the hydrogen bonding environment of W_cat_ and O_w_ in the E-S and E-piP structures, respectively, suggested that protons from W_cat_ are transferred to the side-chain carboxyl oxygen of Asp95 during the reaction. Therefore, we set the new reaction coordinate as Q2 = d(αP − O_3α_) − d(αP − O_w_) + 0.5*[d(H_w_ − O_w_)-d(H_w_ − O_Asp95_)]. In these simulations (Supplementary Movie S2), initial nucleophilic attack leads to formation of a single transition state with a synchronous ∼2.0 Å bond length in both the forming (αP-O_w_) and the breaking (αP-O_3α_) bonds (Figure 6, also similar to the red model in Figure 4A). Decomposition of TS by breaking the αP-O_3α_ bond subsequently creates dUMP and pyrophosphate products. Results also show that proton transfer (PT) between W_cat_ and Asp95 takes place immediately after the bond forming and breaking (generalized electron transfer, ET) step (Supplementary Movie S2).

The TS model of the QM/MM minimizations contains a pentacovalent αP centre, where the phosphorus preserves its former ligands with elongated bond distances (αP − O_5’_ 1.62 Å, αP − O_1α_ 1.54 Å, αP − O_2α_ 1.56 Å, αP − N_3α_ 1.86 Å, to be compared with 1.61 Å, 1.48 Å, 1.52 Å, 1.64 Å, respectively, in the E-S structure), and in addition, it acquires a fifth ligand with 1.88 Å bond length. The five ligands of the αP acquire a trigonal bipyramidal configuration (bond angles of 86–94°, 110–126° and 174°). This structure strongly resembles the predicted trigonal bipyramidal transition state of the associative concerted A_N_D_N_ reaction (predicted distances: equatorial 1.60 Å, axial 1.91 Å, and angles: 90°, 120° and 180°) (54). Although this pentacovalent structure can be well fitted into the observed density maps (Figure 4A), it is unlikely that such a high-energy intermediate would persist long enough to be trapped in a crystallographic experiment, and we therefore interpret this density map as a mixture of E-S and E-piP complexes [cf also (4) for a similar treatment of mixtured complexes].

The QM/MM minimizations provided a rational model for the catalytic reaction; however, they did not clearly show multiple minima along the reaction coordinate, although the experimentally observed E-S and E-piP structures are expected to be associated with different energy levels. Further computational studies with additional techniques would be required to fully resolve this issue.

## CONCLUSIONS

In this study, we describe ‘in situ’ mechanistic and kinetic studies as well as crystallographic visualization of several intermediates for dUTP hydrolysis, also supported by QM/MM computations. These results trace the course of the reaction and create a four-dimensional molecular movie (Supplementary Movie S1). Reaction starts with dUTPase capturing and properly positioning two substrates: dUTP and the catalytic water molecule (W_cat_). W_cat_ is placed near the αP opposite from the leaving group. Hydrolysis is then initiated by nucleophilic attack of W_cat_ on the αP (Figures 3 and 4) along the line of the scissile bond (in-line attack). As a result, a symmetric phosphorane-like transition state structure is formed with significant bond order (∼2.0 Å bond length) in both forming and breaking bonds (Figures 3–6). Bond breaking in the TS is concomitant with inversion of the α-phosphate configuration (Figures 3E and 4C) and pyrophosphate–Mg^2+^ escape. The dUMP product then relaxes by rotation of the α-phosphate group and establishes a configuration similar to the one observed for this moiety in the E-S complex (Figures 3F and 4D). Finally, the enzyme is reset to perform another reaction by favourable replacement of product with fresh substrate. We introduced a novel ^31^P-NMR-based enzyme assay for physicochemical and quantitative kinetic characterization of the enzymatic reaction. Results allow direct structure-guided modelling of the energetically favoured transition state structure and offer the starting point for transition state analogue inhibitor design (86). In this study, the M-PMV dUTPase was used as a model system due to its great crystallization properties. The features of the nucleophilic attack are expected to be similar for other trimeric dUTPases as well, based on the strict conservation of the three-dimensional fold and the side chains within the active site, as well as the current knowledge on other dUTPase mechanistic aspects (cf Introduction). Our study could not provide additional data on the role of the C-terminal arm in the catalysis, as this segment could not be localized in the present structures. A recent study focuses on these questions by using a chemical modification (38). Importantly, the novel post-inversion product structure may propose creative fresh hints for inhibitory compounds. For dUTPase in particular, such antagonists may likely offer potent therapeutics [cf. (5,14,15,24)].

## ACCESSION NUMBERS

3TPN, 3TPS, 3TP1, 3TPY, 3TQ3, 3TQ4, 3TQ5, 3TRL, 3TRN, 3TS6, 3TTA, 3TSL.

## SUPPLEMENTARY DATA

Supplementary Data are available at NAR Online, including [47,87–91].

## FUNDING

Supported by the Hungarian Scientific Research Fund [OTKA K68229, OTKA-A08 CK78646, OTKA NK-84008]; the New Hungary Development Plan [TAMOP-4.2.1/B-09/1/KMR-2010-0002 and Baross ID: 3DSTRUCT, OMFB-00266/2010 REG-KM-09-1-2009-0050]; the Howard Hughes Medical Institutes [#55005628 and #55000342, USA, to B.G.V.]; Bolyai fellowship (to B.A.); [HPMT-CT-20000-00174, EU, to O.B., OTKA NK101072 to A.P.]; and the Intramural Program of the NIDDK, NIH (to O.B. and E.R.). Funding for open access charge: European Molecular Biology Laboratory.

*Conflict of interest statement*. None declared.

## Supplementary Material

Supplementary Data
