# Supplementary material for: Catalytic mechanism of α-phosphate attack in dUTPase is revealed by X-ray crystallographic snapshots of distinct intermediates, 31P-NMR spectroscopy and reaction path modelling
Source: Nucleic Acids Res. 2013 Aug 27;41(22):10542–55. doi: 10.1093/nar/gkt756 (PMC3905902; doi:10.1093/nar/gkt756)
Supplement: Supplementary Data [file supp_41_22_10542__index.html]

Catalytic mechanism of α-phosphate attack in dUTPase is revealed by X-ray crystallographic snapshots of distinct intermediates, 31P-NMR spectroscopy and reaction path modelling — Supplementary Data 

# Catalytic mechanism of α-phosphate attack in dUTPase is revealed by X-ray crystallographic snapshots of distinct intermediates, 31P-NMR spectroscopy and reaction path modelling

## Supplementary Data

files

**Files in this Data Supplement:**

- Supplementary Data - pdf file
- Supplementary Data - wmv file
- Supplementary Data - wmv file
