# Supplementary material for: Catalytic mechanism of α-phosphate attack in dUTPase is revealed by X-ray crystallographic snapshots of distinct intermediates, 31P-NMR spectroscopy and reaction path modelling
Source: Nucleic Acids Res. 2013 Aug 27;41(22):10542–55. doi: 10.1093/nar/gkt756 (PMC3905902; doi:10.1093/nar/gkt756)
Supplement: Supplementary Data [file supp_gkt756_nar-00002-h-2013-File011.pdf]

**Supporting Data to accompany the study entitled:**

# Catalytic mechanism of α-phosphate attack in dUTPase is revealed by X-ray crystallographic snapshots of distinct intermediates, $^{31}$P-NMR spectroscopy and reaction path modeling

Orsolya Barabás[1,2,3,*], Veronika Németh[1], Andrea Bodor[4], András Perczel[4,5], Edina Rosta[6], Zoltán Kele[7], Imre Zagyva[1], Zoltán Szabadka[8], Vince I. Grolmusz[8], Matthias Wilmanns[8] and Beáta G. Vértessy[1,10*]

[1]Institute of Enzymology, Hung. Acad. Sci., Budapest, H-1113 Hungary; [2]Laboratory of Molecular Biology, NIDDK, NIH, Bethesda, MD 20892, USA; [3]EMBL, Heidelberg, D-69117 Germany; [4]Protein Modelling Group MTA-ELTE, Institute of Chemistry, Eötvös Loránd University, Budapest, H-1117 Hungary; [5]Laboratory of Structural Chemistry and Biology, Institute of Chemistry, Eötvös Loránd University, Budapest, H-1117 Hungary; [6]Laboratory of Chemical Physics, NIDDK, NIH, Bethesda, MD 20892, USA; [7]Department of Medical Chemistry, University of Szeged, Hungary, [8]Department of Computer Science, Eötvös Loránd University, Budapest, Hungary, [9]EMBL, Hamburg Outstation, Hamburg, D-22603 Germany, [10]Department of Applied Biotechnology and Food Sciences, Budapest University of Technology and Economics, Budapest, Hungary

* Corresponding authors: Orsolya Barabás, phone: +49 62213878626; fax: +49 62213878306; email: barabas@embl.de, and Beáta G. Vértessy, phone: +36 12793116; fax: +36 14665465; email: vertessy@enzim.hu

## Supplementary Materials and Methods

**Chemicals, protein expression and purification.** α,β-imido-dUTP was obtained from Jena Bioscience, other chemicals of analytical grade purity were from either Merck or Sigma. M-PMV dUTPase was expressed and purified as described previously (1,2) . Enzyme kinetic parameters towards dUTP were measured by the continuous spectrophotometric assay (3,4). α,β-imido-dUTP hydrolysis was followed either by $^{31}$P-NMR (see below) or by separating substrate and product nucleotides with monoQ anion exchange column or with thin layer chromatography (2,5,6). For the later discontinuous assays, aliquots were taken at different time points from crystallization drops or from reaction mixtures containing 5 mg/ml M-PMV NC-dUTPase and 1-15 mM α,β-imido-dUTP in 0.1 M Tris/HCl pH 7.8, 5-15 mM $MgCl_2$, 400 mM Na-acetate, at room temperature.

**Protein Crystallization and Data Collection.** Protein solutions were dialyzed overnight against 20 mM Tris/HCl buffer pH 8.0, containing 200 mM $NH_4Cl$, and 1 mM TCEP. The dUTPase:nucleotide:$Mg^{2+}$ complex solution used for co-crystallization contained 5 mg/ml enzyme, 10 mM $MgCl_2$, and either 1.2 mM α,β-imido-dUTP, 10 mM dUTP or no ligand, respectively. These solutions were mixed with equal volume of reservoir solution (0.1 M Tris/HCl buffer pH 8.5 containing 8-10% PEG8000) in a hanging drop vapour diffusion setup. Hexagonal rod crystals appeared after less than three days of incubation and belonged to space group $P6_3$. All crystals were isomorphous with unit cell parameters ranging from 60.3-61.2 and 63.5-64.5 Å for cell edges a and c, respectively. For data collection, crystals were cryoprotected by addition of 8-10% MPD into the crystallization drops or into separate cryo-soaking drops. Crystals were flash frozen by dipping them into liquid nitrogen at desired time points. Datasets were collected at various synchrotron beamlines at EMBL/DESY (Hamburg, Germany) (Table S1). Data processing and scaling were carried out using MOSFLM (7) and SCALA (8) or XDS (9).

Catalytic competence of the enzyme in the crystalline phase was verified by following α,β-imido-dUTP concentration in the crystallization drops as well as by competition experiments with the native substrate dUTP. For this latter experiment, crystals of wild type dUTPase containing α,β-imido-dUTP were washed with reservoir solution. 10 mM dUTP was added to a fresh droplet of the reservoir solution and hydrolysis was followed by the discontinuous, thin-layer chromatography based activity assay (2).

**Analysing phosphorus-oxygen distances in the PDB.** We searched the Protein Data Bank (PDB) for intermolecular phosphorus-oxygen distances using the following procedures. We considered only entries containing an enzyme (by a regular expression based search for an EC number in the pdb file) with resolution better than or equal to 2.2 angstroms. 12598 entries met these criteria. The ATOM and HETATM records of these entries were scanned for oxygen and phosphorus atoms. Then, a phosphorus-oxygen pair was included in the search if the following four conditions were met: i) the oxygen atom was from water or a standard amino acid, ii) the phosphorus atom was not covalently bonded to the oxygen (there were no CONECT records indicating a covalent bond), iii) the distance between the two atoms was less than 4.5 angstroms, and iv) each atom had occupancy value of 1.0. All P-O contacts meeting these criteria were plotted on Figure S3.

## Supplementary Tables

**Table S1 Crystallographic data collection and refinement statistics**

| Complexes | E-S | | | E-S/E-piP mix | | | E-piP | | | E-P | | |
|---|---|---|---|---|---|---|---|---|---|---|---|---|
| PDB ID | **3TP1** | 3TPN | 3TPS | **3TPY** | 3TQ3 | 3TQ4 | **3TQ5** | 3TRL | 3TRN | **3TS6** | 3TTA | 3TSL |
| Beamline | **X13** | BW7B | BW7B | **X13** | X11 | X11 | **X11** | X11 | X13 | **X13** | BW7B | X13 |
| Wavelength (Å) | **0.8034** | 0.8430 | 0.8430 | **0.8028** | 0.8124 | 0.8128 | **0.8128** | 0.8128 | 0.8031 | **0.8031** | 0.8430 | 0.8034 |
| Resolution (Å) | **20 – 1.60 (1.68 – 1.60)** | 20 – 1.65 (1.75 – 1.65) | 20 – 1.85 (1.95 – 1.85) | **20 – 1.75 (1.77 – 1.75)** | 20 – 1.85 (1.96 – 1.85) | 20 – 1.60 (1.69 – 1.60) | **20 – 1.40 (1.48 – 1.40)** | 20 – 1.80 (1.90 – 1.80) | 20 – 1.83 (1.93 – 1.83) | **20 – 1.84 (1.94 – 1.84)** | 20 – 2.00 (2.10 - 2.00) | 20 – 2.20 (2.31 - 2.20) |
| Space Group | **$P6_3$** | $P6_3$ | $P6_3$ | **$P6_3$** | $P6_3$ | $P6_3$ | **$P6_3$** | $P6_3$ | $P6_3$ | **$P6_3$** | $P6_3$ | $P6_3$ |
| Unit Cell, a/c (Å) | **60.60 / 63.86** | 60.33 / 64.52 | 60.71 / 64.36 | **60.56 / 63.64** | 60.59 / 64.15 | 60.56 / 64.02 | **60.71 / 63.78** | 60.83 / 64.00 | 60.57 / 63.52 | **60.68 / 63.54** | 60.88 / 63.87 | 60.83 / 64.04 |
| Total observations | **47964 (5264)** | 35902 (5604) | 26408 (3820) | **153156 (4928)** | 129700 (20309) | 65104 (7630) | **70791 (8744)** | 38799 (5838) | 39027 (5654) | **32662 (11408)** | 21526 (2947) | 11971 (1548) |
| Unique reflections | **17501 (2371)** | 15802 (2549) | 11407 (1682) | **13399 (438)** | 11478 (1821) | 17233 (2584) | **26148 (4009)** | 12312 (1861) | 11633 (1691) | **11408 (1679)** | 8979 (1239) | 6238 (844) |
| Redundancy | **2.7 (2.2)** | 2.3 (2.2) | 2.3 (2.3) | **11.4 (11.3)** | 11.3 (11.2) | 3.8 (3.0) | **2.7 (2.2)** | 3.2 (3.1) | 3.4 (3.3) | **2.9 (2.8)** | 2.4 (2.4) | 1.9 (1.8) |
| Completeness (%) | **99.2 (99.8)** | 97.9 (98.3) | 98.6 (100) | **99.8 (100)** | 99.8 (99.7) | 97.6 (97.0) | **99.3 (99.8)** | 98.2 (100) | 99.1 (99.2) | **98.3 (99.5)** | 97.8 (98.7) | 90.3 (90.3) |
| I/σ(I) | **17.0 (3.2)** | 15.4 (2.1) | 9.6 (2.1) | **44.8 (4.4)** | 20.2 (5.0) | 13.3 (2.9) | **13.4 (2.6)** | 12.2 (3.1) | 21.5 (5.1) | **17.1 (3.7)** | 11.7 (2.6) | 10.3 (2.6) |
| R-sym (%) | **4.0 (32.0)** | 4.0 (48.8) | 6.7 (51.5) | **3.2 (40.2)** | 7.8 (53.9) | 7.2 (40.3) | **7.1 (41.7)** | 6.3 (46.3) | 3.6 (28.0) | **4.1 (35.9)** | 6.0 (49.3) | 6.3 (28.9) |
| **Refinement** | | | | | | | | | | | | |
| Resolution (Å) | **20 - 1.60** | 20 - 1.65 | 20 - 1.85 | **20 - 1.75** | 20 - 1.85 | 20 - 1.60 | **20 - 1.40** | 20 - 1.80 | 20 – 1.83 | **20 - 1.84** | 20 – 2.0 | 20 - 2.20 |
| Reflections | **16534** | 14564 | 10746 | **12600** | 10863 | 16305 | **24713** | 11657 | 11017 | **10798** | 8493 | 5833 |
| Reflect.- test set | **966** | 822 | 607 | **705** | 614 | 929 | **1385** | 653 | 618 | **610** | 482 | 333 |
| Non-H atoms | **1012** | 1024 | 945 | **1038** | 999 | 1010 | **1002** | 951 | 967 | **957** | 940 | 870 |
| Water molecules | **151** | 120 | 71 | **131** | 99 | 102 | **107** | 102 | 97 | **119** | 96 | 46 |
| R-work (%) | **16.7** | 15.7 | 16.9 | **15.2** | 15.4 | 17.2 | **17.9** | 16.1 | 15.7 | **16.7** | 16.2 | 19.3 |
| R-free (%)[a] | **19.7** | 18.2 | 19.3 | **18.6** | 16.9 | 19.1 | **19.3** | 18.8 | 18.2 | **20.1** | 20.4 | 22.9 |
| Rmsd bond (Å) / angle (°)[b] | **0.019 / 1.96** | 0.020 / 1.98 | 0.020 / 2.04 | **0.018 / 1.96** | 0.019 / 1.81 | 0.019 / 1.95 | **0.020 / 2.03** | 0.020 / 1.83 | 0.018 / 1.81 | **0.019 / 1.99** | 0.019 / 1.89 | 0.016 / 1.70 |
| Mean B | **33.6** | 24.5 | 28.9 | **26.2** | 24.3 | 23.9 | **22.7** | 34.2 | 29.1 | **29.5** | 32.3 | 20.1 |
| B-factor | | | | | | | | | | | | |
| Substrate | **32.6** | 18.1 | 30.0 | **23.2** | 20.4 | 14.1 | **n.a.** | n.a. | n.a. | **n.a.** | n.a. | n.a. |
| product | **n.a.** | n.a. | n.a. | **15.5** | 16.9 | 15.9 | **15.3** | 24.5 | 21.6 | **25.9** | 27.6 | 27.8 |
| $Mg^{2+}$ | **31.6** | 16.4 | 28.5 | **28.1** | 19.0 | 25.4 | **n.a.** | n.a. | n.a. | **n.a.** | n.a. | n.a. |
| Est. coord. err.[c] | **0.076** | 0.079 | 0.104 | **0.091** | 0.092 | 0.075 | **0.053** | 0.096 | 0.096 | **0.109** | 0.133 | 0.197 |

Structures discussed in the main text are in bold. Values in parentheses correspond to the highest resolution shell.[a] Root mean square deviation from ideal/target geometries. [b] Computed for a randomly selected 5% of the data that was excluded from the refinement. [c] Cruickshank's diffraction-component precision index (DPI) for coordinate error..

**Table S2 Conformations of catalytic protein residues as a function of the progress of the reaction.**

| Residue | Role | Observed conformers | Structures represented in |
|---|---|---|---|
| Base (β-hairpin, Tyr98, Ile94) | Uracil and ribose coordination | One conformer in all structures | all |
| Asp95 | $W_{cat}$ binding | Apo-like | E |
| | | Ligand bound | all others |
| Asp42 | $Mg^{2+}$ binding | Apo-like | E, E-piP, E-P |
| | | Nucleotide-$Mg^{2+}$ bound | E-S, E-AI, E-S/E-piP mix |
| Ser80, Gln123 | α-phosphate | One conformer in all structures | all |
| Ser81 | phosphate chain coordination | One conformer in all structures | all |
| Arg120 | phosphate chain coordination | Delocalized | E, E-S, E-AI, E-piP, E-P |
| | | Substrate bound | E-S, E-S/E-piP mix |
| Arg79 | phosphate chain coordination | Delocalized | E |
| | | Nucleotide-$Mg^{2+}$ bound | E-S, E-AI, E-S/E-piP mix |
| | | Product bound (Mg-free) | E-piP, E-P |

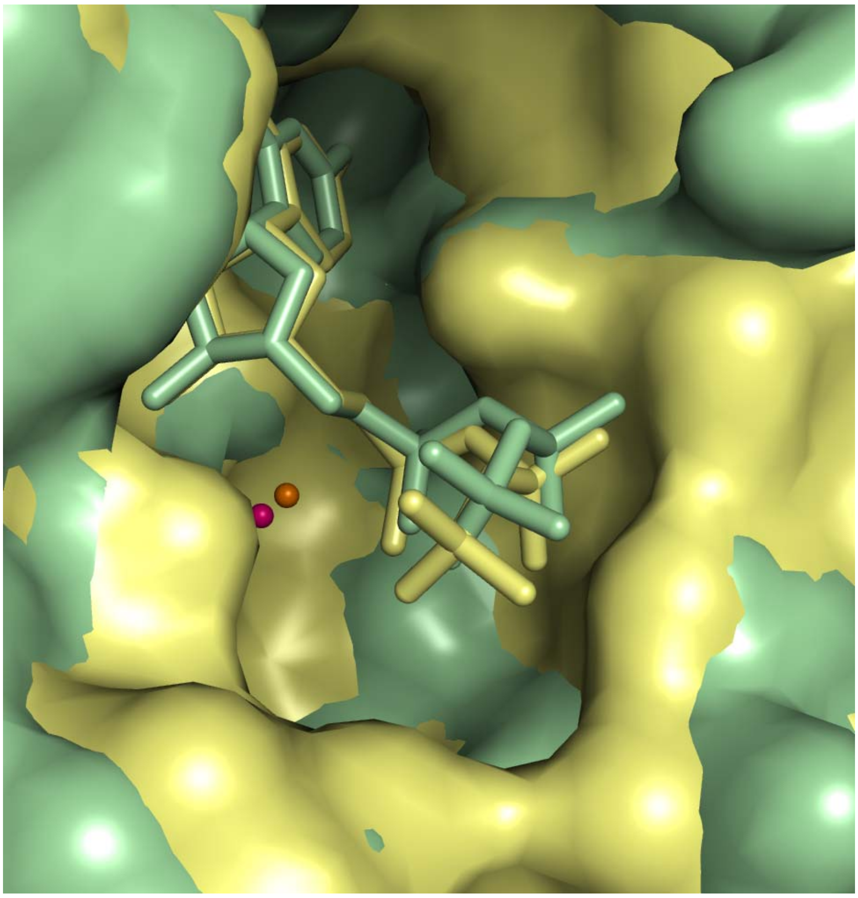

**Figure S1 Superimposed structures of E. coli dUTPase - α,β-imido-dUTP (PDB ID: 1RN8, green) and M-PMV dUTPase complete E-S complexes (PDB ID: 3TP1, yellow).**

Protein surface is shown around the active site, and ligands are represented as sticks. The bound catalytic water molecule is shown in pink for the E. coli dUTPase and orange for the M-PMV dUTPase. Note that the yellow surface (M-PMV dUTPase) covers up the green (E. coli dUTPase) in the phosphate binding region of the active site, while at the base of the pocket the green surface shows up as well. This reveals that the phosphate binding pocket is tighter in the M-PMV enzyme than in the *E. coli* dUTPase, while the base of the active site is similar. Accordingly, the phosphate chain is more folded in the retroviral enzyme structure and the binding site of the catalytic water molecule is very close to the α-phosphorus. The fact that the M-PMV enzyme has a tighter substrate binding cleft may explain why the distance between the αP and the $W_{cat}$ oxygen ($O_w$) (2.52 Å) in the M-PMV dUTPase:substrate:$W_{cat}$ complex is not only shorter than the sum of the van der Waals radii of the two atoms (3.32Å), but also much shorter than that observed in the *E. coli* dUTPase structure (3.6 Å).

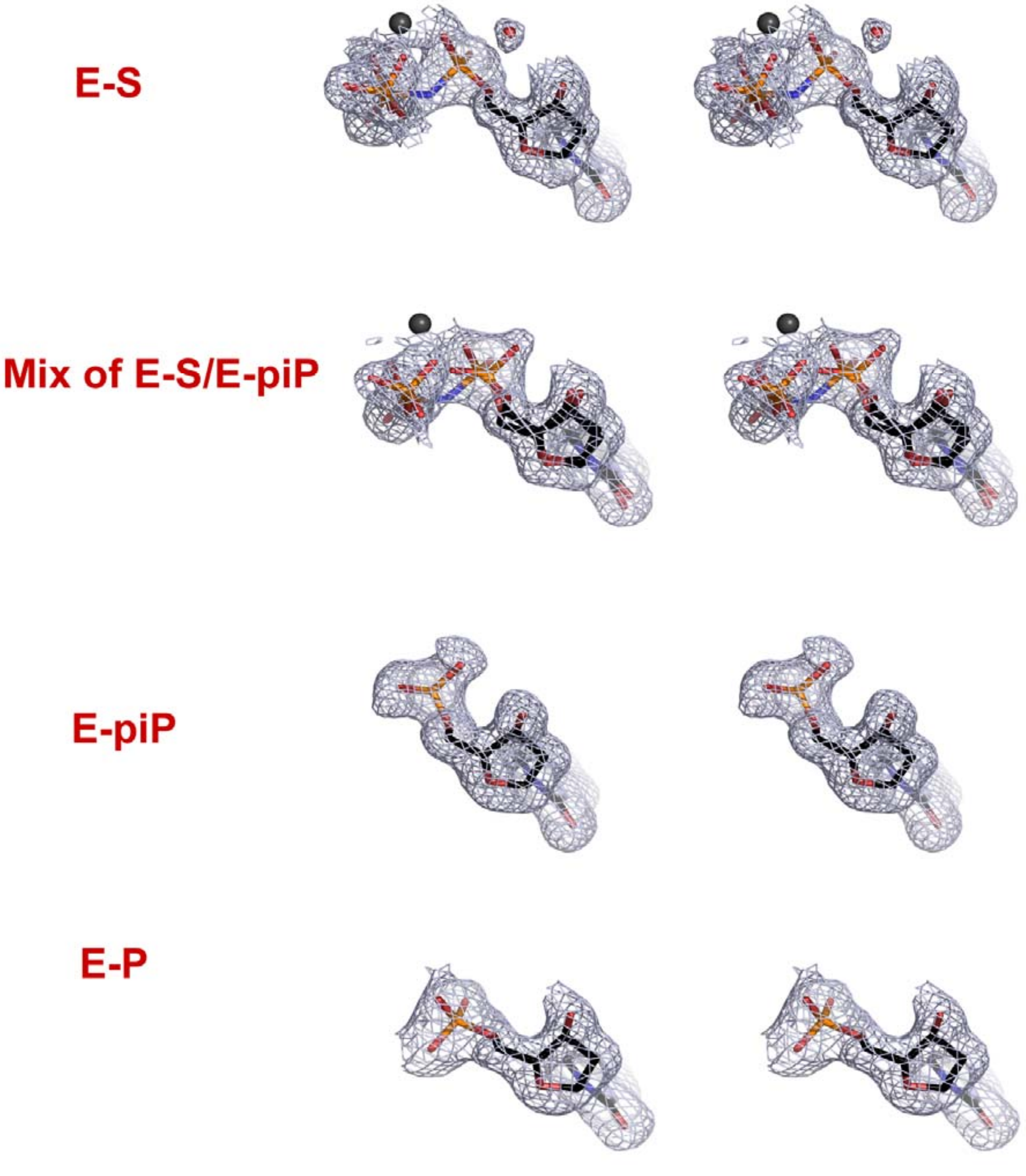


Figure S2. Stereo views of the simulated annealed 2Fo-Fc omit electron density maps (show non Figure 3 in the main text) are presented for reactants and products in representative structures at various sigma levels: E-S - 1.3σ, E-S/E-piP mix - 1.0σ, E-piP - 1.5σ and E-P - 1.7σ.

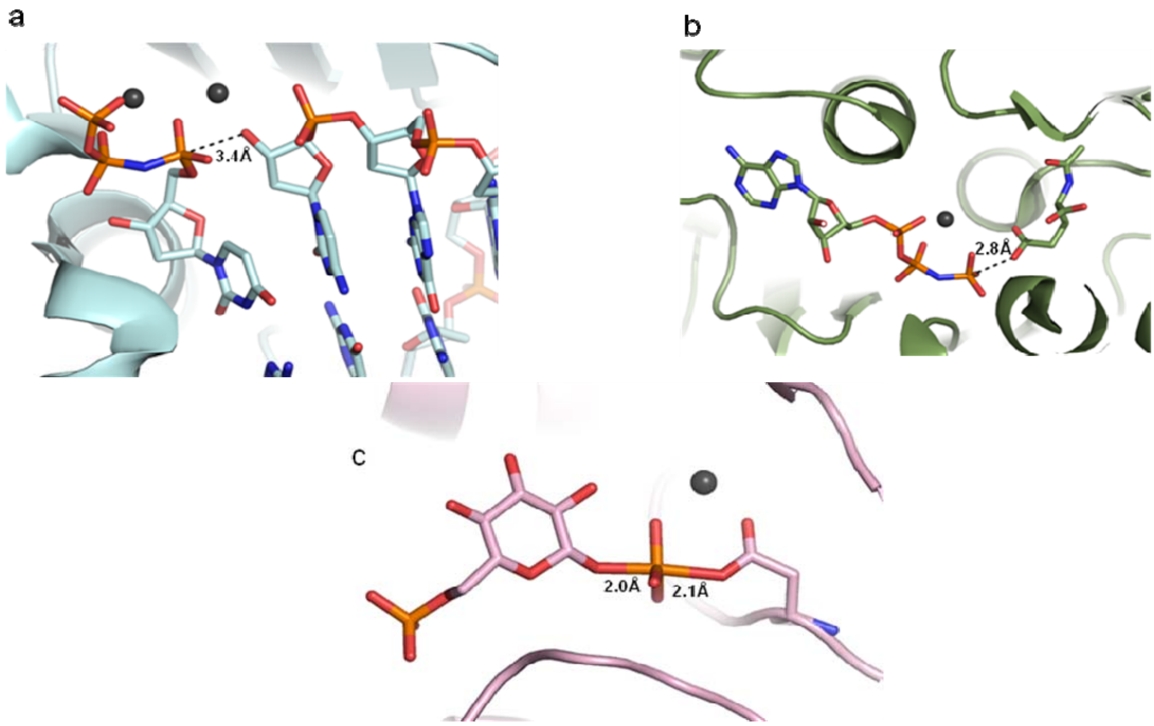


**Figure S3 Examples of crystal structures visualizing a reaction intermediate.** DNA polymerase β (PDB ID: 2FMS, a) represents an E-S complex, acetylglutamate kinase (PDB ID: 1GS5, b) an E-AI structure, while the phosphoglucomutase (PDB ID: 1O08, c) structure was suggested to capture the "high-energy" penta-coordinated phosphorane intermediate. Interatomic distances between the nucleophilic attacker oxygen and the phosphorus center are also shown.

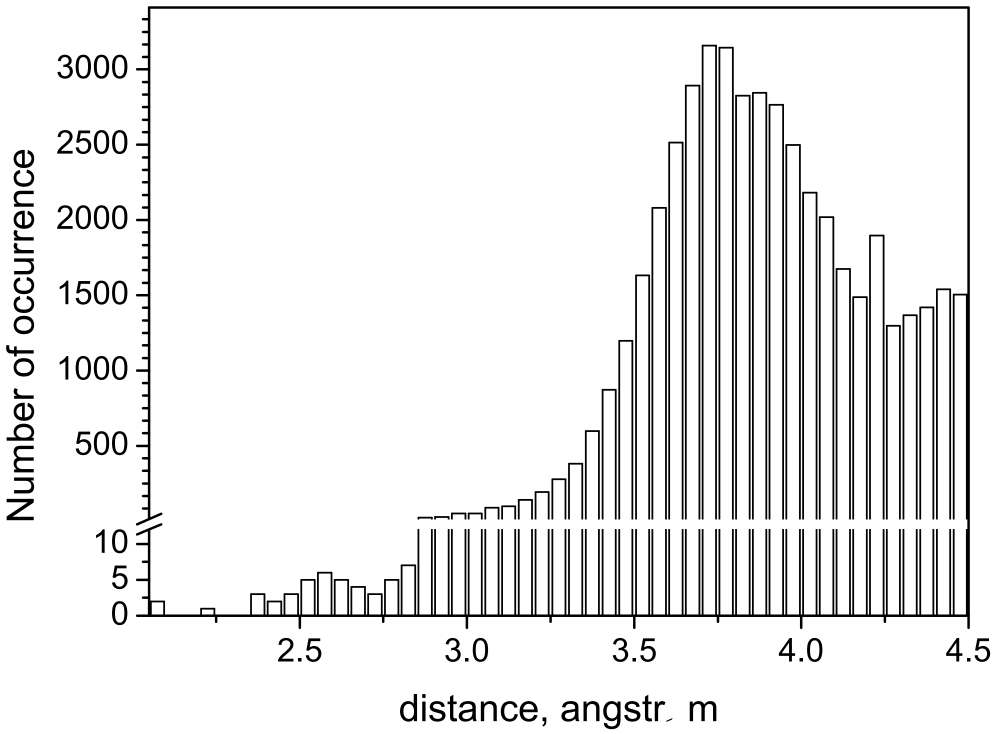


**Figure S4 Distribution of distances between non-bonded phosphorus and oxygen atoms in enzyme structures.** The graph shows the number of intermolecular phosphorus-oxygen contacts with certain P-O distances in the Protein Data Bank (PDB). Note that a low but appreciable number of P-O pairs are significantly shorter than the sum of the two atoms' van der Waals radii (rvdW(P) + rvdW(O) = 3.32Å).

**Supplementary Movies**

**Movie S1 Molecular movie of dUTP hydrolysis by the dUTPase enzyme.**

The movie shows a model for dUTP hydrolysis by the M-PMV dUTPase enzyme including approach of the nucleophile water molecule, formation of a pentavalent transition state, inversion of the α-phosphate configuration and rotation of the product α-phosphate to a stable end-conformation. The animation was created in Pymol using linear morphing between experimentaly observed crystal structures. The color code is as in Figure 1c, the $Mg^{2+}$-ion is shown in magenta.

**Movie S2 QM/MM results trace the catalytic pathway.**

The movie shows the structures of the QM region obtained from the QM/MM minimizations for the hydrolysis reaction by M-PMV dUTPase. The animation illustrates the reaction pathway: the nucleophile water attacks the phosphate group, a symmetric pentavalent transition state is formed, the α-β phosphate bond breaks and the water proton is transferred to the Asp95 residue.

**Supplementary References**


1. Nemeth-Pongracz, V., Barabas, O., Fuxreiter, M., Simon, I., Pichova, I., Rumlova, M., Zabranska, H., Svergun, D., Petoukhov, M., Harmat, V. *et al.* (2007) Flexible segments modulate co-folding of dUTPase and nucleocapsid proteins. *Nucleic Acids Res*, **35**, 495-505.
2. Barabas, O., Rumlova, M., Erdei, A., Pongracz, V., Pichova, I. and Vertessy, B.G. (2003) dUTPase and nucleocapsid polypeptides of the Mason-Pfizer monkey virus form a fusion protein in the virion with homotrimeric organization and low catalytic efficiency. *J Biol Chem*, **278**, 38803-38812.
3. Vertessy, B.G. (1997) Flexible glycine rich motif of Escherichia coli deoxyuridine triphosphate nucleotidohydrolase is important for functional but not for structural integrity of the enzyme. *Proteins*, **28**, 568-579.
4. Kovari, J., Barabas, O., Takacs, E., Bekesi, A., Dubrovay, Z., Pongracz, V., Zagyva, I., Imre, T., Szabo, P. and Vertessy, B.G. (2004) Altered active site flexibility and a structural metal-binding site in eukaryotic dUTPase: kinetic characterization, folding, and crystallographic studies of the homotrimeric Drosophila enzyme. *J Biol Chem*, **279**, 17932-17944.
5. Vertessy, B.G., Persson, R., Rosengren, A.M., Zeppezauer, M. and Nyman, P.O. (1996) Specific derivatization of the active site tyrosine in dUTPase perturbs ligand binding to the active site. *Biochem Biophys Res Commun*, **219**, 294-300.
6. Vertessy, B.G., Zalud, P., Nyman, P.O. and Zeppezauer, M. (1994) Identification of tyrosine as a functional residue in the active site of Escherichia coli dUTPase. *Biochim Biophys Acta*, **1205**, 146-150.
7. Leslie, A.G.W. (1992) Recent changes to the MOSFLM package for processing film and image plate data. *Joint CCP4 + ESF-EAMCB Newsletter on Protein Crystallography*, **26**.
8. CCP4. (1994) The CCP4 suite. Programs for protein crystallography. *Acta Crystallogr D Biol Crystallogr*, **50**, 760-763.
9. Kabsch, W. (1993) Automatic processing of rotation diffraction data from crystals of initially unknown symmetry and cell constants. *J. Appl. Cryst.*, **26**, 795-800.
